# Endogenous retroviral long terminal repeats as host gene promoters in normal and cancer cells

**DOI:** 10.1186/1742-4690-11-S1-P132

**Published:** 2014-01-07

**Authors:** Artem Babaian, Dixie L Mager

**Affiliations:** 1 Terry Fox Laboratory, British Columbia Cancer Agency, Canada; 2 Dept. of Medical Genetics, University of British Columbia, Vancouver, BC, Canada

## 

The human genome contains nearly 400,000 sequences related to retroviruses that have accumulated due to ancient infections of the germ line and subsequent fixation during evolution. Most of these endogenous retroviral sequences (ERVs) currently exist as solitary long terminal repeats (LTRs), the product of recombination between LTRs of the integrated proviral form. Since retroviral LTRs naturally contain transcriptional promoters and enhancers, these sequences have great potential to impact regulation of individual genes and gene regulatory networks. Numerous cases of LTRs serving as promoters for human genes have been described by our group and others, and some examples will be presented. While such co-option of LTRs as regulatory gene modules indeed occurs in normal cells, particularly in placenta or in early development, transcriptional activity of most endogenous LTRs is epigenetically suppressed in somatic tissues, likely as a host defense against unregulated transcription. Cancer cells represent an abnormal epigenetic environment where LTRs and other classes of transposable elements (TEs) are often hypomethylated, leading to their transcriptional activation. This activation could result in abnormal, cancer-specific expression of nearby genes. To study this phenomenon, we are analyzing whole transcriptome data of cancers specifically to identify gene deregulation due to transcriptional activity of LTRs or other TEs. Our results suggest that the regulatory potential of these sequences is often used by cancer cells, providing one avenue to up-regulate genes. Thus, while some LTRs/TEs have been co-opted to serve in normal gene expression, the same regulatory qualities can be exploited in carcinogenesis.

